# Hantavirus Infection with Marked Sinus Bradycardia, Taiwan

**DOI:** 10.3201/eid0806.010340

**Published:** 2002-06

**Authors:** Yuan-Hong Liu, Jyh-Hsiung Huang, Po-Ren Hsueh, Kwen-Tay Luh

**Affiliations:** * National Taiwan University Hospital, Taipei, Taiwan; †En Chu Kong Hospital, Taiwan; ‡ Department of Health, Taipei, Taiwan

To the Editor: Hantaviruses are enveloped RNA viruses belonging to the family *Bunyaviridae* (*1*,*2*), for which a number of species have been identified, including the Hantaan, Seoul, Puumala, Dobrava-Belgrade, and Sin Nombre viruses (*1*,*2*). Each hantavirus is associated with a specific rodent reservoir (*1*,*2*). *Hantaan virus*, found throughout northeastern Asia, causes a life-threatening illness known as hemorrhagic fever with renal syndrome (HFRS). Main symptoms and signs of HFRS are fever, myalgia, severe vascular leakage with ascites and retroperitoneal edema and pain (abdominal, loin, or headache), shock, acute renal failure, proteinuria and hematuria, thrombocytopenia, and bleeding complications (*3*). *Seoul virus*, found worldwide, and *Puumala virus*, found in Scandinavia and Eastern Europe, cause mild forms of HFRS. *Sin Nombre* *virus*, found in the United States, causes hantavirus pulmonary syndrome, which is characterized by increased pulmonary capillary permeability and pulmonary edema and can progress to severe respiratory distress syndrome and shock as a result of low cardiac output (*4*,*5*).

Despite the fact that HFRS is frequently reported in People’s Republic of China, no indigenous cases of HFRS have been reported in Taiwan. Previous serologic studies found that the Seoul strain is endemic in the areas of Taiwan and two isolated islands nearby, Kinmen and Matzu; in contrast, in the People’s Republic of China, the Hantaan and Seoul strains concurrently predominate (*6*,*7*).

Our patient, a 38-year-old man, had onset of sore throat, headache, cough, myalgia, and intermittent fever (up to 38.3°C) on February 2, 2001. A resident of Matzu for more than 30 years, he had traveled to the People’s Republic of China 3 months before the symptoms began. Laboratory tests at a local hospital showed thrombocytopenia (58,000/mL) and leukopenia (3,800/mL). Because his symptoms persisted, he was transferred to the National Taiwan University Hospital on February 7, 2001. Initial tests there showed a temperature of 36.4°C, heart rate 74 beats/min, and respiratory rate 18/min; there was no skin rash. The rest of the physical examination was normal. He had a platelet count 73,000/µL; leukocytes 5,670/µL with 59.1% segments, 19.8% lymphocytes, and 18.2% monocytes; urea nitrogen 7.4 mg/dL; and creatinine 0.94 mg/dL. Urinalysis showed proteinuria (300 mg/dL). His chest radiography was normal. Abdominal ultrasound showed a fatty liver.

After admission, the patient’s laboratory values gradually improved and his proteinuria subsided. He had no fever. On February 10, 2001, he had marked sinus bradycardia (as low as 33 beats/min) and became fatigued. His blood pressure was 120–130/70–80 mmHg. No abnormal serum electrolytes, urea nitrogen, creatinine, creatine kinase, and troponin-I were noted. Echocardiogram showed normal atrium and ventricle size, good left ventricle contractility, and small amount of pericardial effusion. His heart rate gradually increased. He was discharged on February 15, 2001, without event.

A substantial increase of serum immunofluorescent immunoglobulin (Ig) G titers (1:640 on February 6; 1:5120 on February 19, 2001) and positive IgM titers of 1:80 against hantavirus antigen (Seoul type) confirmed that this virus was responsible for the illness.

A few reports of hantavirus infection with cardiac involvement have been published. A case report by Chun and Godfrey showed right atrium dilation with diffuse atrial hemorrhage, interstitial edema, and vascular congestion without surrounding myocardial fibers and conduction system involvement in a 19-year-old soldier who died from epidemic (Korean) hemorrhagic fever, sinus tachycardia, paroxysmal supraventricular tachycardia, and congestive heart failure (*8*). Marked sinus bradycardia (as low as 34 beats/min) in a patient with a severe form of hemorrhagic fever with renal syndrome (acute renal failure) has been reported (*9*). However, this finding was not observed in patients with mild cases of the disease.

The possibility that our patient acquired the infection during his travel to the People’s Republic of China 3 months earlier is extremely low because of the length of the incubation period (typical incubation period 4–28 days) (*10*) and the different hantavirus strains prevalent in the People’s Republic of China (*6*). Although viral genetic sequence data from the patient and rodents in Matzu were not available in this study, our patient was infected with the Seoul strain, which is highly seroprevalent in rodents in Matzu (*6*,*7*).

In summary, this case was probably the first indigenous case of hantavirus infection in Taiwan. Its characteristics suggest that marked sinus bradycardia should be included as a protean manifestation of hantavirus.
